# GraphDTI: A robust deep learning predictor of drug-target interactions from multiple heterogeneous data

**DOI:** 10.1186/s13321-021-00540-0

**Published:** 2021-08-11

**Authors:** Guannan Liu, Manali Singha, Limeng Pu, Prasanga Neupane, Joseph Feinstein, Hsiao-Chun Wu, J. Ramanujam, Michal Brylinski

**Affiliations:** 1 Division of Electrical and Computer Engineering, Louisiana State University, Baton Rouge, LA 70803 USA; 2 Department of Biological Sciences, Louisiana State University, Baton Rouge, LA 70803 USA; 3 Center for Computation and Technology, Louisiana State University, Baton Rouge, LA 70803 USA; 4 Department of Computer Science, Brown University, Providence, RI 02902 USA

**Keywords:** Drug–target interactions, Protein–protein interaction network, Drug perturbed gene expression, Feature selection, Multi-layer perceptron, Machine learning, Deep learning, GraphDTI

## Abstract

Traditional techniques to identify macromolecular targets for drugs utilize solely the information on a query drug and a putative target. Nonetheless, the mechanisms of action of many drugs depend not only on their binding affinity toward a single protein, but also on the signal transduction through cascades of molecular interactions leading to certain phenotypes. Although using protein-protein interaction networks and drug-perturbed gene expression profiles can facilitate system-level investigations of drug-target interactions, utilizing such large and heterogeneous data poses notable challenges. To improve the state-of-the-art in drug target identification, we developed GraphDTI, a robust machine learning framework integrating the molecular-level information on drugs, proteins, and binding sites with the system-level information on gene expression and protein-protein interactions. In order to properly evaluate the performance of GraphDTI, we compiled a high-quality benchmarking dataset and devised a new cluster-based cross-validation protocol. Encouragingly, GraphDTI not only yields an AUC of 0.996 against the validation dataset, but it also generalizes well to unseen data with an AUC of 0.939, significantly outperforming other predictors. Finally, selected examples of identified drugtarget interactions are validated against the biomedical literature. Numerous applications of GraphDTI include the investigation of drug polypharmacological effects, side effects through offtarget binding, and repositioning opportunities.

## Introduction

Comprehensive knowledge of system-level interactions between small organic molecules and their macromolecular targets is of paramount importance to modern drug discovery. The vast majority of drug targets are proteins whose biological functions are determined by their interactions with other molecular species in a cell [1]. Because of the central roles of proteins in numerous biological processes, any changes in their structures and functions, caused by mutations and other factors, often lead to a disease state [2]. Pharmacotherapeutics are designed to bind to these disrupted proteins in order to mitigate disease conditions [3]. Since drug molecules usually bind to specific sites formed by the concave regions of target protein surfaces, drug-target interactions (DTIs) can, in principle, be investigated using the complex structures of proteins in their ligand-bound conformational states. In the absence of experimentally determined complex structures, theoretical models can be constructed by molecular docking methods to study putative, low-energy binding modes of drugs bound to their protein targets [4, 5].

Inverse virtual screening (IVS) is a traditional method to identify drug targets for small molecules. Structure-based IVS techniques employ molecular docking to screen a ligand against a database of proteins in order to find a subset of binding sites that are putative targets for the query molecule [6]. An example of a docking-based method is TarFisDock [7], a webserver utilizing the docking program DOCK [8] to dock small molecules into either the Potential Drug-Target Database containing 698 protein structures [9], or a custom list of target sites provided by a user. Candidate targets are then ranked based on the interaction energy computed with van der Waals and electrostatic terms. Encouragingly, TarFisDock predicted 10 putative targets for 4 H-tamoxifen and 12 for vitamin E, many of which are experimentally verified targets. Another docking-based IVS program is idTarget employing a divide-and-conquer docking approach combined with quantum chemical charge models and robust regression-based scoring functions [10]. To constrain the search space for a putative binding site for a query ligand, a large docking box, initially covering the entire surface of a target protein, is constructed and then its size is dynamically reduced to smaller grids. idTarget conducts screens against nearly all protein structures present in the Protein Data Bank [11] and has been demonstrated to be able to reproduce known off-targets of drugs and drug-like compounds.

Nonetheless, the molecular actions of many drugs may be difficult to determine solely based on their interactions with single targets because the phenotypes of many complex diseases often depend on numerous molecular interactions through which the information in a cell is passed from one protein to another [12]. In order to account for this intricacy of the molecular basis of complex diseases, the study of molecular mechanisms of drugs and their system-level effects often involves the analysis of the structures of protein-protein interaction (PPI) networks [13]. Indeed, it was demonstrated that putative drug targets can be identified in a PPI network based on several topological features, such as the modularity, the coreness, and the eccentricity [14]. Further, drug targets can be distinguished from those proteins that are not targets for small molecules based on their degree, 1-N index, clustering coefficient, shortest distance to drug targets, average distance to drug targets, betweenness, and topological coefficient [15]. Interestingly, among the top 200 proteins ranked by their topological features, as many as 94 are either known drug targets in DrugBank [16] or putative targets supported by the biomedical literature.

In addition to the analysis of PPI networks, potential drug targets can be identified from the differential gene expression profiles of various cell lines. For instance, the activatory and inhibitory targets of drug candidates can be predicted by comparing gene expression profiles collected for cell lines perturbed with the chemical treatment, gene knockdown, and gene overexpression [17]. Direct correlation methods typically analyze correlation coefficients between differential gene expression profiles measured for the chemical treatment and either a gene knockdown or a gene overexpression. These coefficients can be used as predictive scores not only to identify highly correlated drug-protein pairs, but also to suggest a drug mechanism of action. Essentially, a high correlation between gene expression profiles for the chemical treatment and the gene knockdown indicates the inhibition, whereas the activation is predicted when the chemical treatment correlates with the gene overexpression profiles. In addition to the direct correlation methods, predictive models for individual target proteins can also be constructed using joint learning techniques. These predictive models learn shared similarities between gene knockdown and gene overexpression signatures in order to identify the activatory and inhibitory targets for small molecules. Importantly, selected interactions in drug-target-disease association networks predicted by comparing gene expression profiles for 1,124 drugs, 829 target proteins, and 365 human diseases have been validated in vitro.

The Library of Integrated Network-based Cellular Signatures (LINCS), the largest repository of gene expression profiles collected for numerous perturbagens and cell lines [18], is often used in studies focused on the drug target identification. For instance, a method employing the tensor decomposition-based unsupervised feature extraction utilized the LINCS data to identify the so-called “inferred genes” and “inferred compounds” as being associated with the dose dependence [19]. In order to predict target proteins for small molecules, “inferred genes” can be compared to a single-gene perturbation using the gene list enrichment analysis tool Enrichr [20]. Interestingly, as many as 195 genes identified as common drug targets are significantly enriched with molecular function terms related to protein-ligand binding according to the Gene Ontology database [21]. Another approach first identifies sets of deregulated genes by small molecules by comparing gene expression profiles from drug-treated and control cell lines, and then calculates a proximity score for each protein in the human PPI network with a new measure called the local radiality (LR) [22]. Encouragingly, as many as 22 % of known drug targets were found in the 1st percentile of protein lists ranked by the LR.

Many contemporary studies focused on DTIs utilize large, complex, and highly heterogeneous datasets including biological and biochemical networks, transcriptomics, bioassay and screening data, etc. Not surprisingly, machine learning methods have become invaluable tools in computational biology to overcome the challenge of inferring the knowledge from these exponentially growing repositories [23]. Two distinct groups of techniques are currently employed to predict DTIs with supervised machine learning, similarity- and feature-based approaches [24]. Methods belonging to the former group typically first compute two similarity matrices, one for drugs and another for targets, which are then used to predict DTIs with various kernel functions, such as nearest neighbor [25], kernel regression [26], and bipartite local models [27]. Nonetheless, the major drawback of similarity-based methods is that these algorithms often have difficulties predicting novel interactions from unseen data. On the other hand, feature-based approaches employ feature vectors representing individual instances as drug molecular structures and some information on target proteins. These feature vectors are then often used with traditional machine learning methods, such as support vector machines [28], decision trees [29], and random forests [30]. Feature-based approaches not only consider the information for drugs and proteins separately, but also suffer from a high computational complexity due to the high dimensionality of feature vectors.

More recently, deep neural networks (DNNs) have become the state-of-the-art predictors across numerous fields, including natural language processing [31], image processing [32], and big data analytics [33]. Not surprisingly, DNNs are commonly employed as robust classifiers in the field of computational biology to extract information from the complex biological data. For instance, a convolutional neural network (CNN) was utilized to classify ligand-binding sites [34], and a deep belief network (DBN) was applied to analyze and predict the toxicity of drug candidates [35]. Because of their remarkable versatility, deep learning methods are well suitable to predict DTIs as well. An example is recently developed DeepDTIs, which employs a DBN with extended connectivity fingerprints and protein sequence composition descriptors as features [36]. A similar algorithm, DeepLSTM, utilizes a long short-term memory (LSTM) architecture as the DTI predictor against multiple datasets [37]. Other methods, such as DeepConv-DTI [38] and DeepDTA [39], use CNNs to predict DTIs. DeepConv-DTI works with the descriptors of protein sequences and the Morgan fingerprints of drugs, while DeepDTA consists of two separate CNNs to predict drug-target affinities from raw protein sequences and the SMILES strings of drugs. Encouragingly, the performance of DeepConv-DTI is 0.80 in terms of the area under the curve (AUC), while the mean squared error (MSE) for predictions made by DeepDTA is 0.26.

Despite a promising progress in DTI prediction, important challenges remain. Many previous models employ either the information on drugs and proteins, combined or separately, or drug-perturbed gene expression profiles and PPI networks to predict DTIs. Therefore, one apparent advancement is to better integrate multiple heterogeneous data to infer interactions between drugs and their targets with a higher sensitivity and a lower false positive rate. Another future direction is to more carefully design validation protocols for supervised machine learning methods. In many studies reported to date, training and validation subsets were created by randomly splitting DTI datasets. Because of various redundancies present in these datasets in terms of drug and protein similarities, this procedure may lead to an inflated performance and poor capabilities of the trained classifiers to generalize to unseen data. In order to address these problems, we developed GraphDTI, a new method integrating multiple heterogeneous data to predict DTIs. Biological data utilized by GraphDTI comprise target protein sequences, drug chemical structures, the structures of drug binding sites, and the information obtained from drug-perturbed gene expression profiles. The effective representations of DTIs are derived from local graphs centered on drug targets in the human PPI network. A feature selection procedure is deployed to reduce the risk of overfitting when training the DNN model used as a classifier to predict DTIs. To mitigate the problem of redundancy in biological datasets, not only a new cluster-based split protocol is used to conduct cross-validation benchmarks, but also the trained machine learning model is ultimately applied to an independent testing dataset in order to properly evaluate the generalizability of GraphDTI to unseen data. In comparative benchmarking calculations against several other algorithms, we demonstrate that GraphDTI offers an unparalleled performance in large-scale DTI prediction.

## Results and discussion

### System-level data representation and integration

**Fig. 1:**
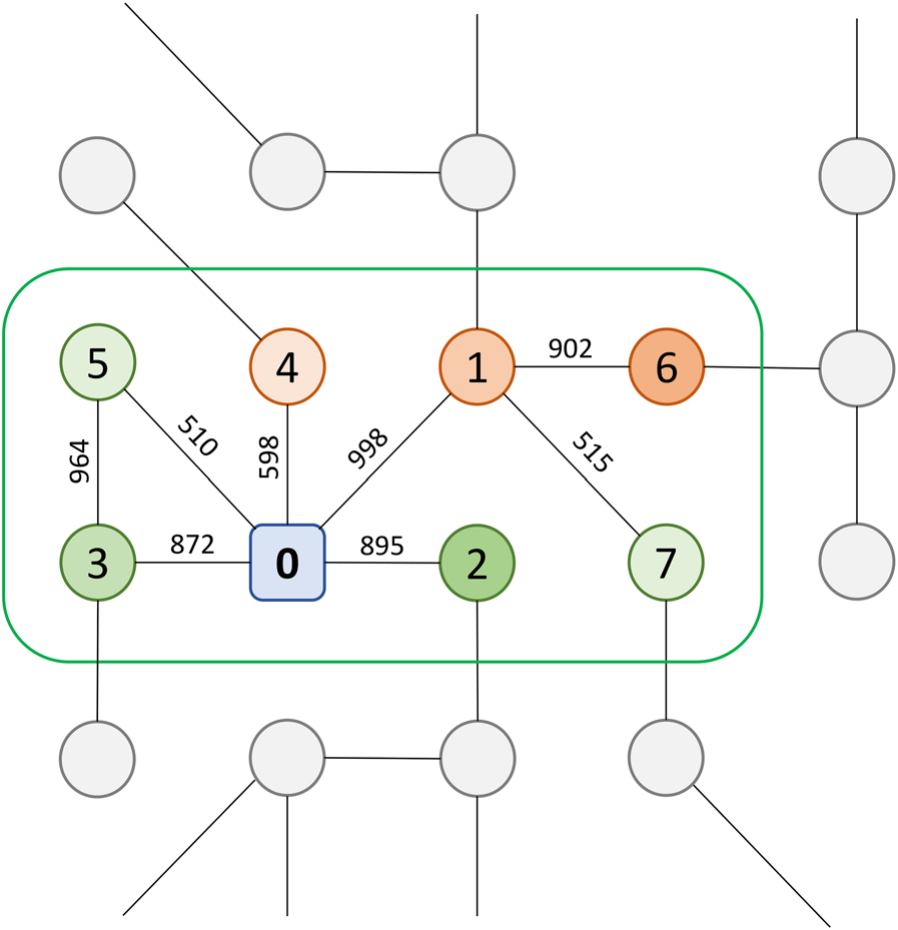
Schematic representation of the local network environment for a target protein. The target is shown as a rounded square and other proteins in the network are circles. A green outline encloses the local environment for the target node comprising *N* top neighbors ranked by their distance to the target node (*N* is set to 7 in this simple example). Numbers inside nodes correspond to ranks by the distance, while numbers along edges are the confidence values for biological interactions between individual proteins. Nodes in the local environment are colored according to their differential gene expression values (green – upregulated, red – downregulated) with the transparency level depending on the magnitude of up- and downregulation

The vast majority of drug candidates developed by conventional target-based discovery approaches do not perform well in clinical trials due to either a reduced efficacy or unexpected adverse effects [40]. To address these issues, the paradigm in drug discovery has shifted from the concept of “one gene, one drug, one disease” to a system-level approach in order to account for the enormous complexity of biological systems involving the information propagation through numerous molecular interactions in a cell and the simultaneous effects of pharmacotherapy on multiple biological processes [41]. In particular, transcriptomic profiles provide invaluable data capturing the system-level effects of drug candidates in biological cells at the outset of drug discovery [40]. Combined with the PPI network information, drug-perturbed differential gene expression profiles help understand how drug binding to molecular targets alters biological processes to produce a particular phenotype [22]. In GraphDTI, an undirected, weighted subgraph containing a central node corresponding to the target (labeled 0 in Fig. 1) with multiple connected nodes representing interacting proteins, is extracted from the entire human PPI network. Each edge is assigned a weight computed as the reciprocal of the confidence score for the interaction between two proteins in the STRING database (numbers along the edges in Fig. 1). Nodes in the subgraph are then ranked in an ascending order according to the length of their shortest paths to the target. This representation captures the local network environment of a given target node to properly propagate the drug-perturbed differential gene expression information in machine learning.

### GraphDTI architecture

**Fig. 2:**
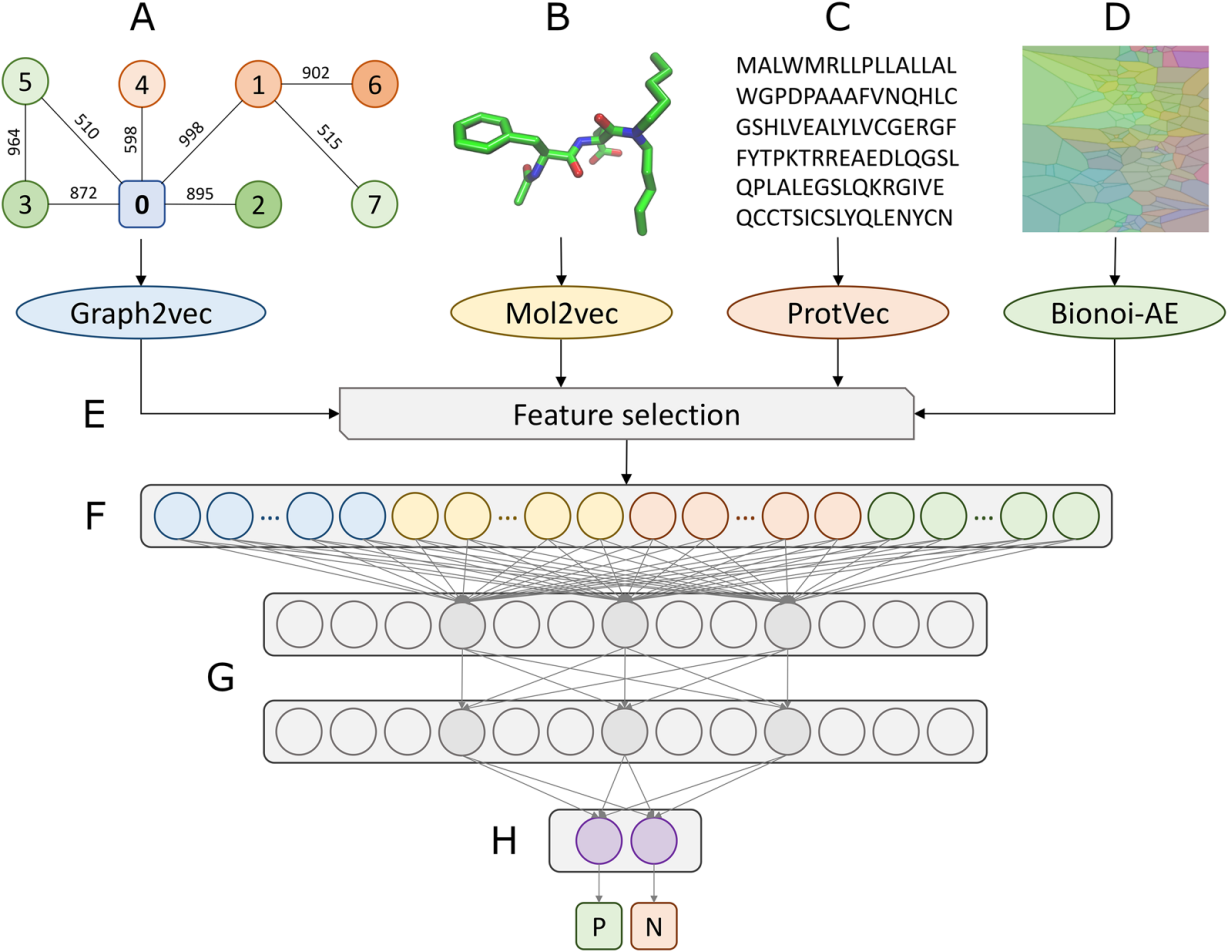
Flowchart of GraphDTI. The input to GraphDTI comprises four feature vectors, **A** a local network environment for the target protein encoded with Graph2vec, **B** a drug chemical structure encoded with Mol2vec, **C** a target protein sequence encoded by ProtVec, and **D** the structural and physicochemical properties of a binding site encoded with Bionoi-AE. **E** A feature selection is employed prior to the input layer in order to reduce the dimensionality of the feature vector. **F**) An input layer concatenating network environment (blue), drug (yellow), protein (red), and binding site (green) feature vectors. **G** Two hidden layers with selected connections for three neurons colored in dark gray. **H** An output layer consisting of two neurons to estimate the probability of the drug-target interaction (P—positive, N—negative)

The overall architecture of GraphDTI is depicted in Fig. 2. In addition to the vector representation of a local graph centered on the target protein extracted from the human PPI network encoded with Graph2vec (Fig. 2A), the input data also contain the vector representations of a drug structure encoded with Mol2vec (Fig. 2B), a protein sequence encoded with ProtVec (Fig. 2C), and a drug-binding site in the target protein encoded with the Bionoi autoencoder (Bionoi-AE, Fig. 2D). Subsequently, a feature selection procedure based on the permutation feature importance is applied prior to the input layer in order to reduce the dimensionality of the feature vector mitigating the risk of overfitting (Fig. 2E). The input layer comprising features selected from local network environment (blue), drug (yellow), protein (red), and drug binding site (green) descriptors (Fig. 2F) is followed by two hidden layers, each containing 128 neurons (Fig. 2G). At the end, an output layer composed of two neurons (Fig. 2H) evaluates the probabilities of a given drug-target instance to be positive (P) and negative (N).

### Feature optimization for the local network environment

**Fig. 3:**
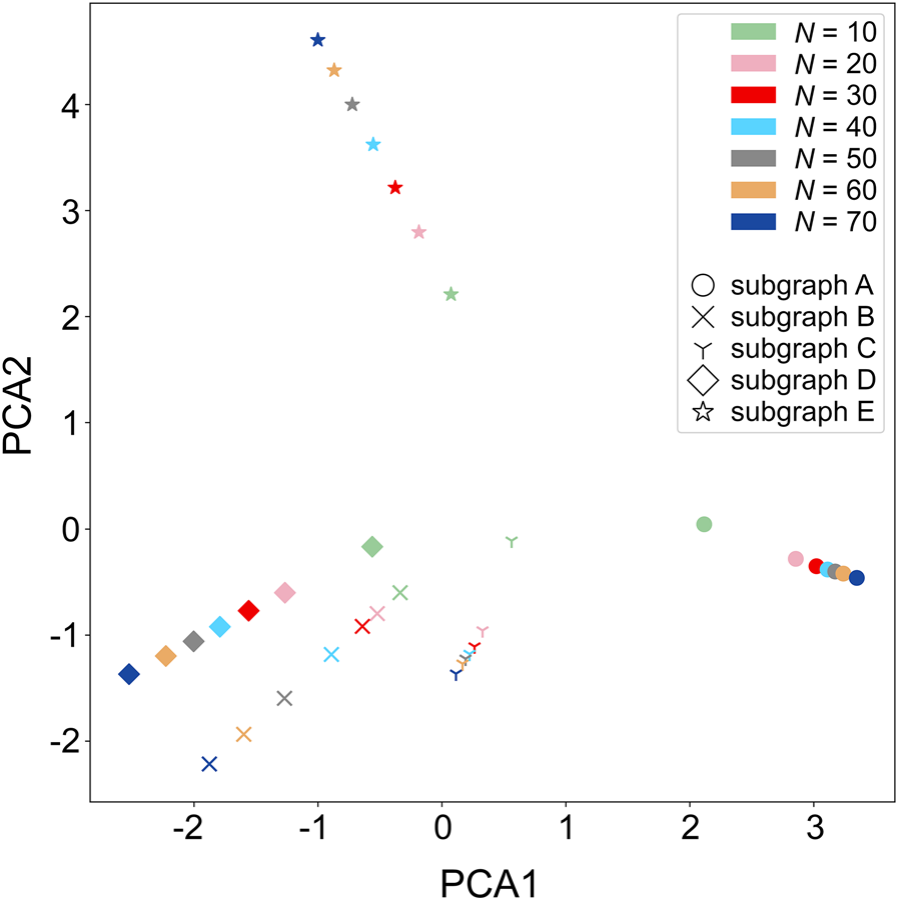
Visualization of sub-graph embeddings for the target local environment. The scatter plot was created by reducing the number of dimensions with the Principal Component Analysis (PCA) of five different subgraphs (represented by different maker shapes), each with several different sizes. The size is defined as the number of highly confident neighbors of a target node (*N*) increasing from 10 to 70 (shown in different colors)

The first optimization of the data representation in GraphDTI is to select the optimal number of nodes in the local network environment centered on a given target protein. In Fig. 3, the Principal Component Analysis [42] is employed to visualize five different subgraphs, represented by various marker shapes and labeled A-E, and seven different configurations, created using a different number of connected nodes *N* ranging from 10 to 70, shown in various colors. As expected, distances between 5 subgraphs in the low-dimensional space tend to increase with the increasing values of *N* indicating that larger local graphs should yield a better discrimination in machine learning.

Next, in order to determine the optimal size of local networks centered on protein targets, a quantitative analysis is conducted by evaluating the classification performance of a multilayer perceptron (MLP) trained on 20,000 instances randomly sampled from the GraphDTI dataset. The MLP model utilizes the same framework as GraphDTI (shown in Fig. 2), except that the number of neurons for the input layer is 600 (300 drug features and 300 local network features). Table 1 reports AUC values for a classification by the MLP model, 5-fold cross validated on network embeddings computed for varying *N* values. The MLP model yields the highest mean AUC score of 0.994 ± 0.001 when *N* is set to 50. Thus, in all subsequent calculations, local network environments for drug targets in GraphDTI are represented by graph embeddings calculated for 50 proteins interacting with the target node in the human PPI network.

**Table 1:** Optimization of the size of the target local environment in the PPI network

*N*	AUC
10	0.978 ±0.003
20	0.985 ±0.002
30	0.989 ±0.002
40	0.991 ±0.002
50	0.993 ±0.001
60	0.985 ±0.002
70	0.985 ±0.002

The Area Under the Curve (AUC) measures the classification performance of the MLP model employing drug and local network embeddings in 5-fold cross-validation against the GraphDTI dataset. Graph embeddings for target nodes are calculated for a number of highly confident neighbors of a target node (*N*) increasing from 10 to 70

### Feature selection with permutation feature importance

**Fig. 4:**
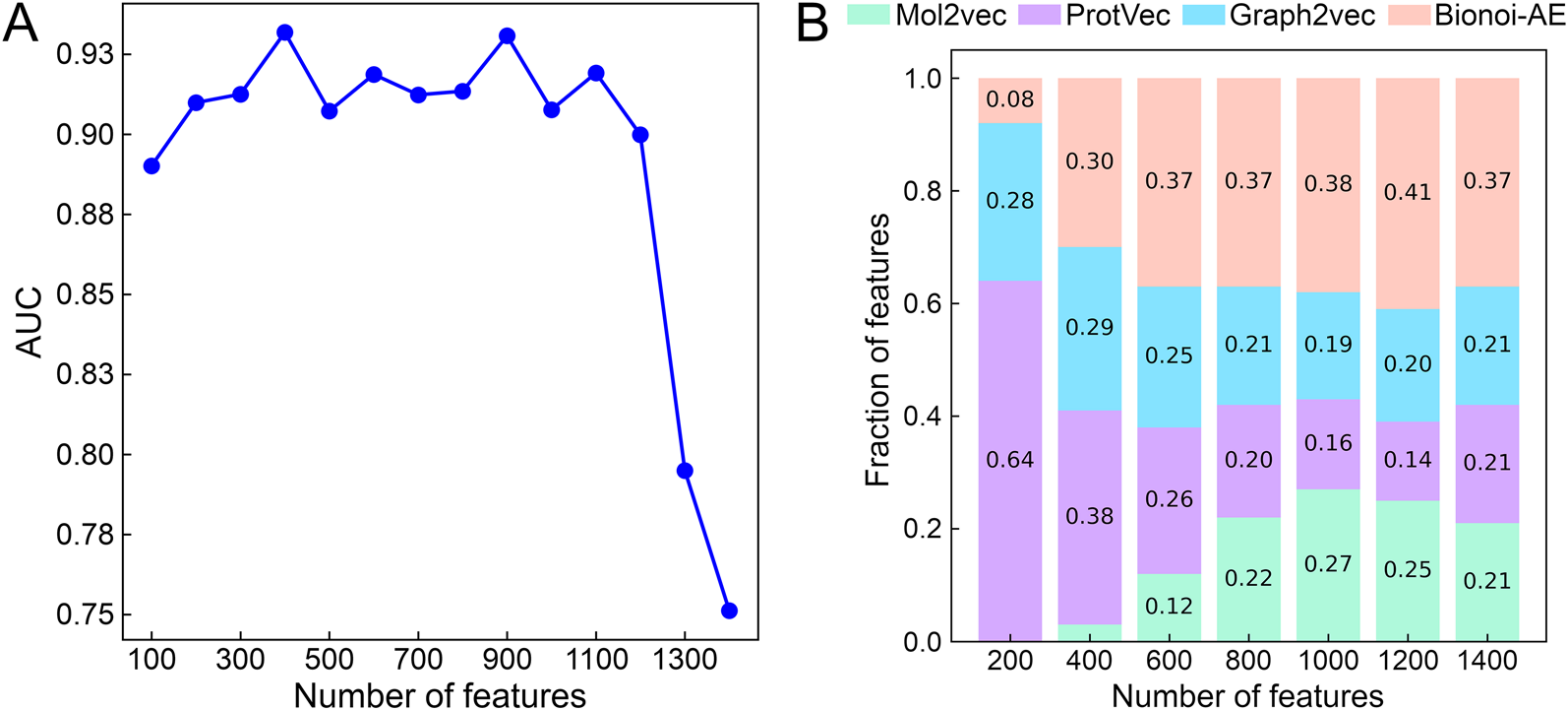
Feature selection with permutation feature importance. **A** The area under the curve (AUC) calculated for a varying number of features selected by the permutation feature importance algorithm against the PubChem BioAssay dataset. **B** The composition of feature vectors changing with the vector length. Feature vectors comprise four groups of features calculated for drugs with Mol2vec (green), proteins with ProtVec (purple), local network environments with Graph2Vec (light blue), and binding sites with Bionoi-AE (orange)

In order to mitigate the effects of overfitting and to reduce the computational complexity, the optimal feature vector is determined by a feature selection procedure based on the importance scores of individual features [43]. Briefly, all 1412 features, comprising 300 drug, 300 protein, 512 drug binding site and 300 local network features, are first ranked in a descending order based on their importance scores estimated with the permutation feature importance algorithm. Next, the classification performance of the MLP model, pre-trained on the GraphDTI dataset, against the PubChem BioAssay dataset is calculated for a different number of the ranked features. In Fig. 4, we evaluate the AUC scores and the composition of feature vectors varying in size. Figure 4A shows that the MLP model yields low AUC scores for feature vectors shorter than 200 because a low-dimensional feature space is insufficient for the model to perform well against unseen data. It also does not generalize well to unseen data for feature vectors longer than 1200 due to the overfitting problem [44]. The MLP model achieves the highest AUC of 0.932 when the feature vector size is set to 400. Figure 4B shows that the composition of feature vectors depends on their size with protein features dominating short vectors, and drug and local network features becoming more prominent in longer vectors. The composition of a 400-dimensional vector yielding the highest classification accuracy is 3 % drug, 38 % protein, 29 % drug binding site, and 30 % local network features. This feature vector is employed in GraphDTI in all subsequent calculations.

### Visualization of the machine learning model

**Fig. 5:**
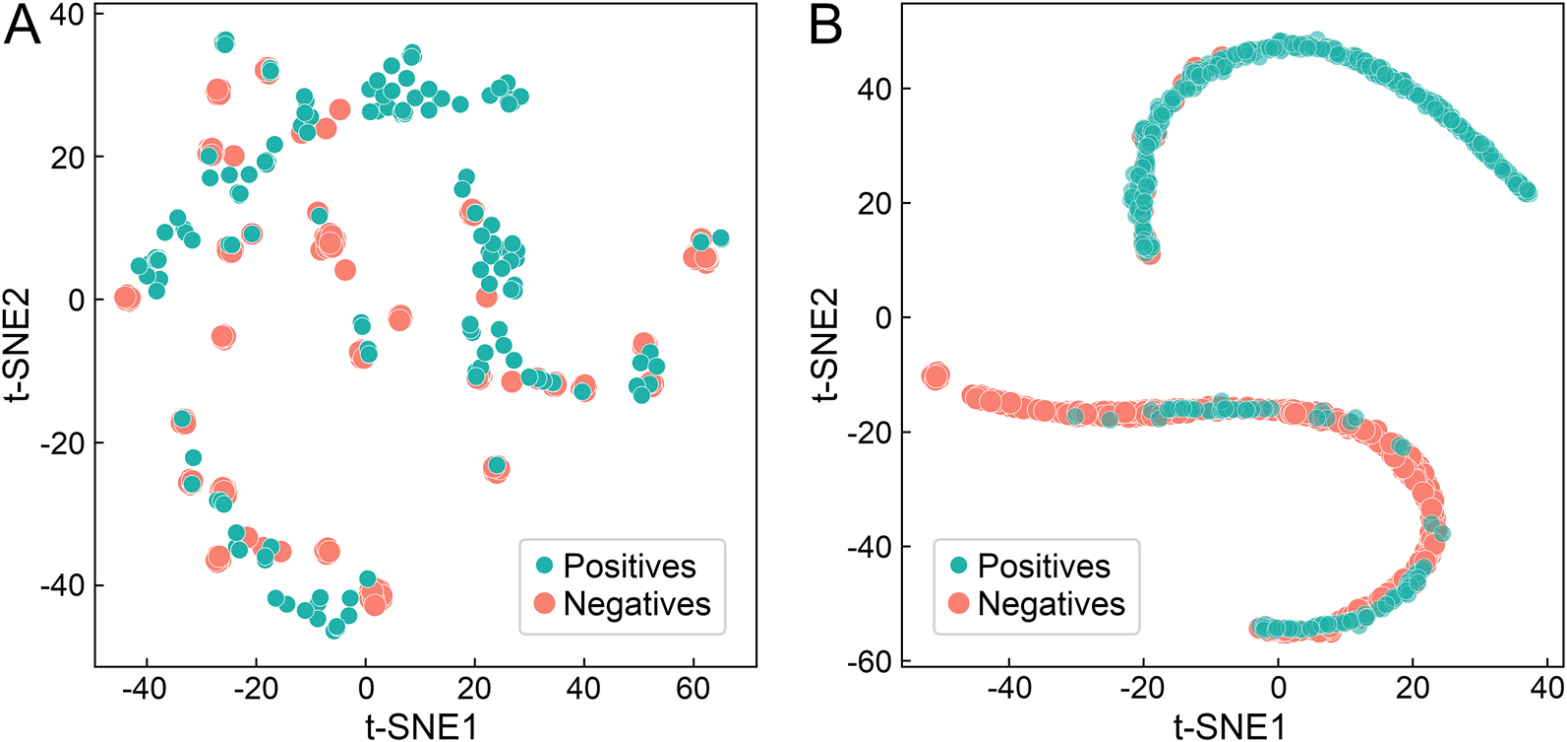
Separation of input features and output-layer embeddings in a low-dimensional space. The T-distributed Stochastic Neighbor Embedding (t-SNE) technique is applied to 500 positive (teal) and 500 negative (salmon) instances randomly selected from the PubChem BioAssay dataset. Dimensionality reduction is conducted for **A** 400-dimensional input feature vectors and **B** output-layer embeddings prior to the softmax activate function

T-distributed stochastic neighbor embedding (t-SNE) is a non-linear dimensionality reduction strategy developed to visualize high-dimensional datasets while minimizing the information loss [45]. Figure 5 shows the visualization of 500 positive (teal) and 500 negative (salmon) instances from the PubChem BioAssay dataset with t-SNE. A dimensionality reduction applied to 400-dimensional feature vectors optimized with the permutation feature importance algorithm is presented in Fig. 5A, whereas Fig. 5B shows the t-SNE visualization of output-layer embeddings prior to the softmax activate function of the pre-trained MLP model. Although 400 important features of positive instances noticeably overlap with those of negative instances, the output-layer embeddings of the MLP model actually separate into two groups, one containing predominantly positive instances and the other composed of mostly negative instances. This analysis indicates that GraphDTI should prove effective in the prediction of DTIs from unseen data.

### Performance of DTI predictors in a random-split cross-validation

**Fig. 6:**
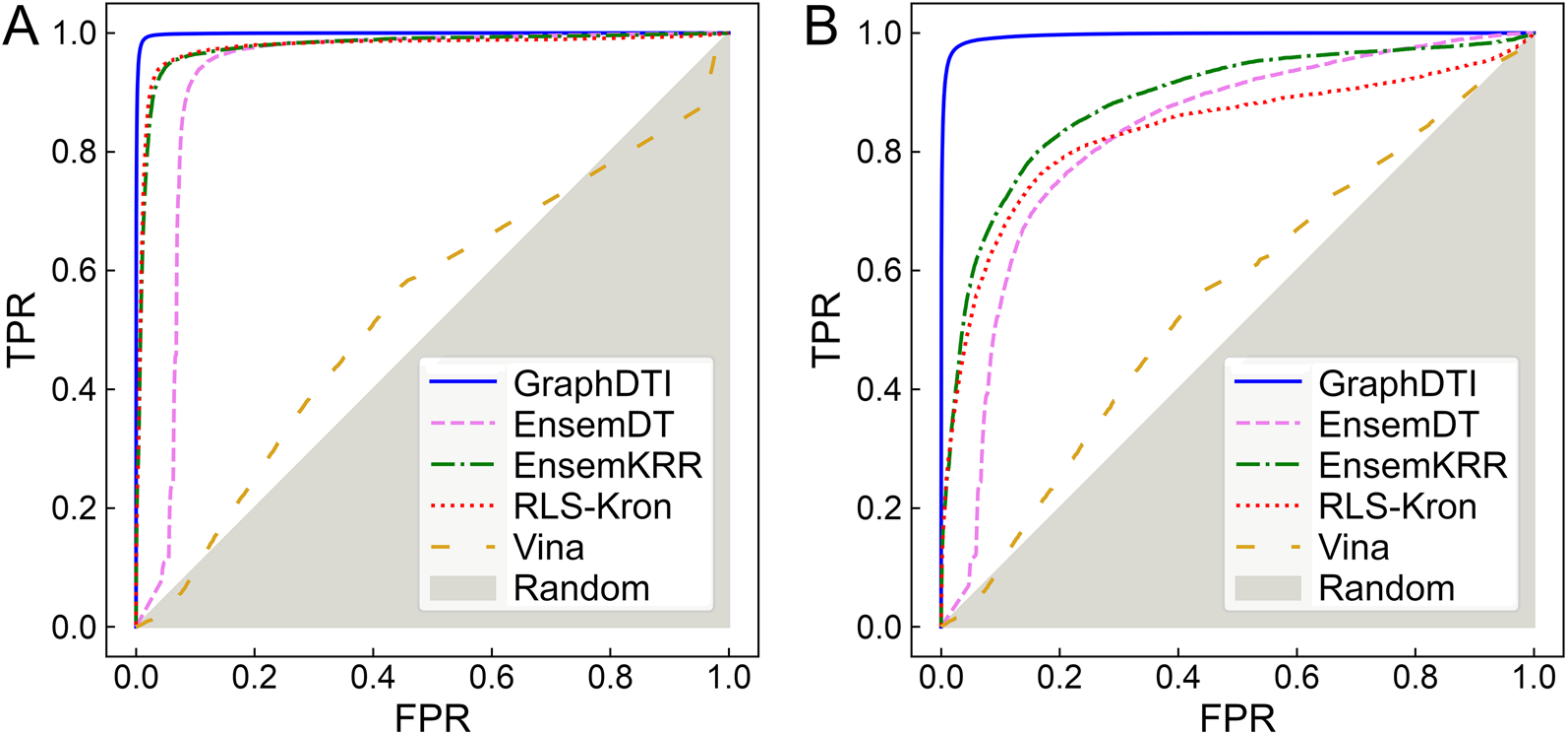
Cross-validated performance of algorithms to predict DTIs. Receiver Operating Characteristic (ROC) plots showing the true positive rate (TPR) against the false positive rate (FPR) are calculated for **A** random-split and **B** cluster-based cross-validation benchmarks against the GraphDTI dataset. The performance of several DTI predictors is presented, GraphDTI (solid blue lines), EnsemDT (dashed pink lines), EnsemKRR (dashed-dotted green lines), RLS-Kron (dotted red lines), and Vina (dashed yellow line). The gray area corresponds to the performance of a random classifier

The performance of GraphDTI is compared to that of three other machine learning methods, EnsemDT, EnsemKRR, and RLS-kron [46], as well as an approach employing molecular docking with AutoDock Vina [5]. EnsemDT is a feature-based algorithm utilizing the Decision Tree, a commonly used machine learning model for classification problems. The other two machine learning methods are similarity-based. EnsemKRR employs RLS-avg base learner [47] with the Kernel Ridge Regression (KRR) classifier. The classification is performed according to the average of two scores calculated separately for the drug kernel and the target kernel. RLS-kron is a similar algorithm utilizing the KRR classifier, however, rather than the average, the prediction score is the Kronecker product of drug and target kernels. The performance of DTI predictors is evaluated with the Receiver Operating Characteristic (ROC) analysis in Fig. 6 with the corresponding AUC values reported in Table 2.

**Table 2:** Performance of algorithms to classify drug–target interactions

Algorithm	GraphDTI dataset		PubChem Bioassay dataset
	Random-split	Cluster-based	
GraphDTI	0.999 ±0.0004	0.996 ±0.0036	0.939
EnsemDT	0.924 ±0.0903	0.824 ±0.0972	0.597
EnsemKRR	0.977 ±0.0029	0.885 ±0.0365	0.488
RLS-Kron	0.976 ±0.0035	0.834 ±0.0393	0.465
Vina	0.534 ±0.0044	0.551 ±0.0372	–

The Area Under the Curve (AUC) measures the classification performance against the GraphDTI dataset, cross-validated with random-split and cluster-based protocols, and the PubChem Bioassay dataset containing unseen data

We first present the results obtained from a 10-fold cross-validation against the GraphDTI dataset randomly split into training and validation subsets. Figure 6A and the second column in Table 2 show that GraphDTI yields a nearly perfect classification performance with an AUC of as high as 0.999. EnsemDT, EnsemKRR, and RLS-kron also perform remarkably well when a protocol based on the random split of data is employed. In contrast to methods employing machine learning, inverse virtual screening with AutoDock Vina has an AUC of only 0.534 demonstrating that this method has rather poor capabilities to predict DTIs against the GraphDTI dataset. Although similar random-split protocols are commonly used to benchmark DTI predictors, the performance of classifiers employing supervised learning methods is likely overestimated on account of a possible overlap between training and validation subsets. Splitting data randomly into folds may result in interactions involving similar drugs and proteins to be assigned to training and validation subsets making it easier to achieve a high classification accuracy.

### Clustering drugs and their molecular targets

In order to address the issue of overlapping data and to properly evaluate the generalizability of DTI predictors, we developed a cluster-based cross-validation protocol ensuring that instances assigned to different folds are distinct from one another. Specifically, 90,353 drug-protein instances in the GraphDTI dataset were clustered with the *k*-medoids algorithm, which is applicable to data partitioning in the Euclidean space [48]. The resulting clusters were evaluated with the Silhouette coefficient (SC) because it provides a convenient measure to evaluate a cohesion, the similarity of an object to its own cluster, against a separation, the dissimilarity of an object to other clusters [49]. SC ranges from − 1 to 1 with higher values indicating that objects are well matched to their own clusters and different from objects belonging to other clusters. Because the *k*-medoids algorithm has a certain randomness, it does not always converge to the same solution. Thus, for a given number of clusters, the data partitioning is repeated 50 times and the mean SC values with the corresponding standard error are computed.

**Fig. 7:**
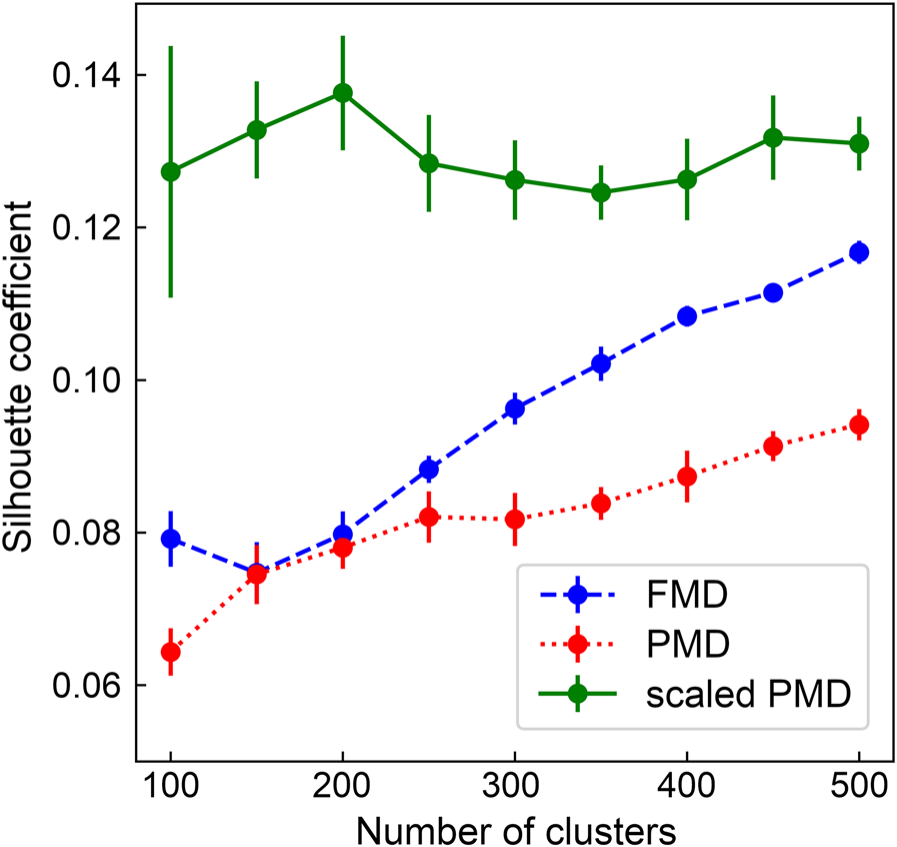
Optimization of the number of clusters for cross-validation. Silhouette coefficient values are calculated for the GraphDTI dataset partitioned with the *k*-medoids algorithm into a varying number of clusters. Three measures of distances between drug-protein pairs are used in the dataset clustering, the Feature Match Distance (FMD, blue), the Perfect Match Distance (PMD, red), and the scaled PMD (green). For a given number of clusters a mean value (circles) with the corresponding error (vertical bars) are plotted

In Fig. 7, we compare the consistency within clusters of data obtained with three distance metrics for drug-protein pairs, the Feature Match Distance (FMD), the Perfect Match Distance (PMD) [50], and the scaled PMD. Using the scaled PMD consistently yields the highest cluster consistency compared to the other distance metrics, for instance, SC values for 200 clusters are 0.080 ±0.003, 0.078 ±0.003 and 0.138 ±0.008 for FMD, PMD and the scaled PMD, respectively. Therefore, we selected the scaled PMD as the best distance measure for the *k*-medoids algorithm with the optimal number of clusters of 200. Next, the resulting 200 clusters were randomly merged into 10 folds for cross-validation. This protocol essentially minimizes similarities between folds in the drug-target space not only making the GraphDTI dataset more challenging for DTI predictors, but also reducing the risk of overfitting in supervised machine learning.

### Performance of DTI predictors in a cluster-based cross-validation

The performance of GraphDTI, EnsemDT, EnsemKRR, RLS-kron, and AutoDock Vina using a 10-fold cluster-based cross-validation is evaluated with the ROC analysis in Fig. 6B with the corresponding AUC values reported in the third column of Table 2. Encouragingly, GraphDTI maintains its high performance in these more challenging benchmarks with an AUC of 0.996. In contrast, the performance of EnsemDT, EnsemKRR, and RLS-kron is notably lower compared to that obtained using the random-split cross-validation protocol. These results indicate that GraphDTI should have high capabilities to generalize to unseen data, whereas the other machine learning methods are going to suffer from overfitting problems. As expected, the performance of AutoDock Vina, which is not a supervised learning method, is independent of the assignment of instances to cross-validation folds.

### Performance of DTI predictors against unseen data

**Fig. 8:**
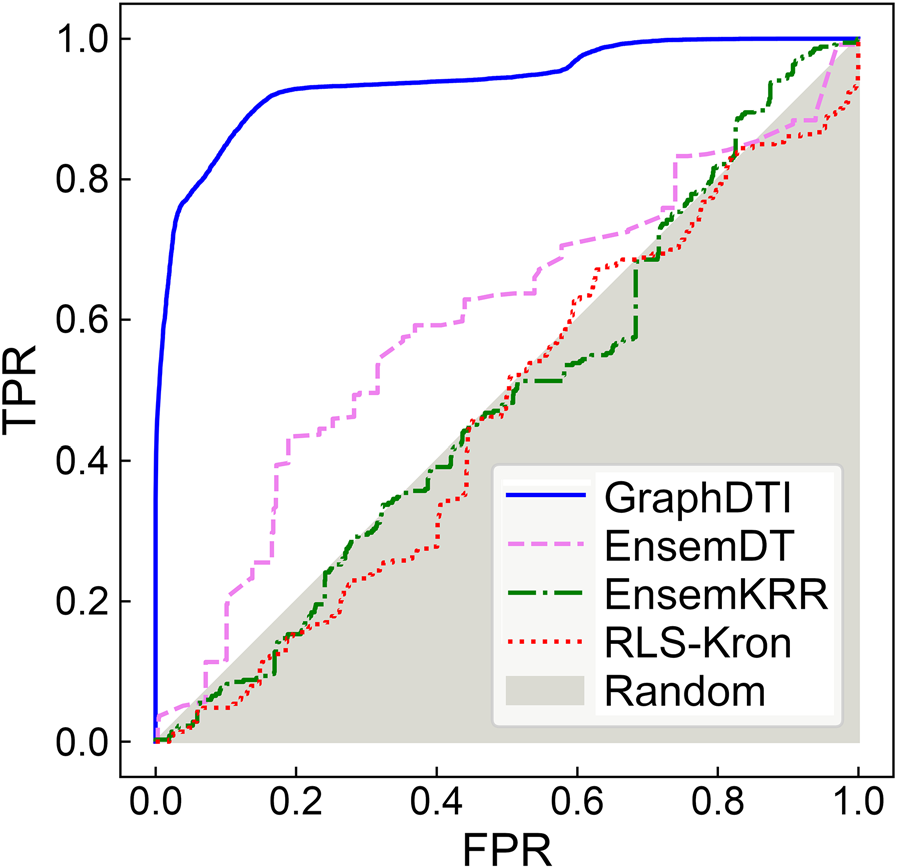
Performance of algorithms to predict DTIs against unseen data. Receiver Operating Characteristic (ROC) plots showing the true positive rate (TPR) against the false positive rate (FPR) are calculated for machine learning models pre-trained on the GraphDTI dataset and applied to classify unseen instances from the PubChem BioAssay dataset. The performance of several DTI predictors is presented, GraphDTI (solid blue lines), EnsemDT (dashed pink lines), EnsemKRR (dashed-dotted green lines), and RLS-Kron (dotted red lines). The gray area corresponds to the performance of a random classifier

Although, the cluster-based cross-validation protocol can help reduce the overlap between training and validation instances, it should always be mandatory to evaluate the performance of DTI predictors against unseen data. On that account, we tested all machine learning methods against an independent dataset compiled from the PubChem BioAssay database [51] with models pretrained on the GraphDTI dataset. The resulting ROC plots are presented in Fig. 8 with the corresponding AUC values reported in the last column of Table 2. As expected, GraphDTI yields the highest AUC score of 0.939, whereas the other machine learning approaches give AUC values around 0.5 demonstrating that, in contrast to GraphDTI, these programs do not have capabilities to generalize to unseen data. In the subsequent sections, we validate several DTIs confidently predicted by GraphDTI in PubChem BioAssay and GraphDTI datasets against the biomedical literature.

### Pharmacology of fasudil

**Fig. 9:**
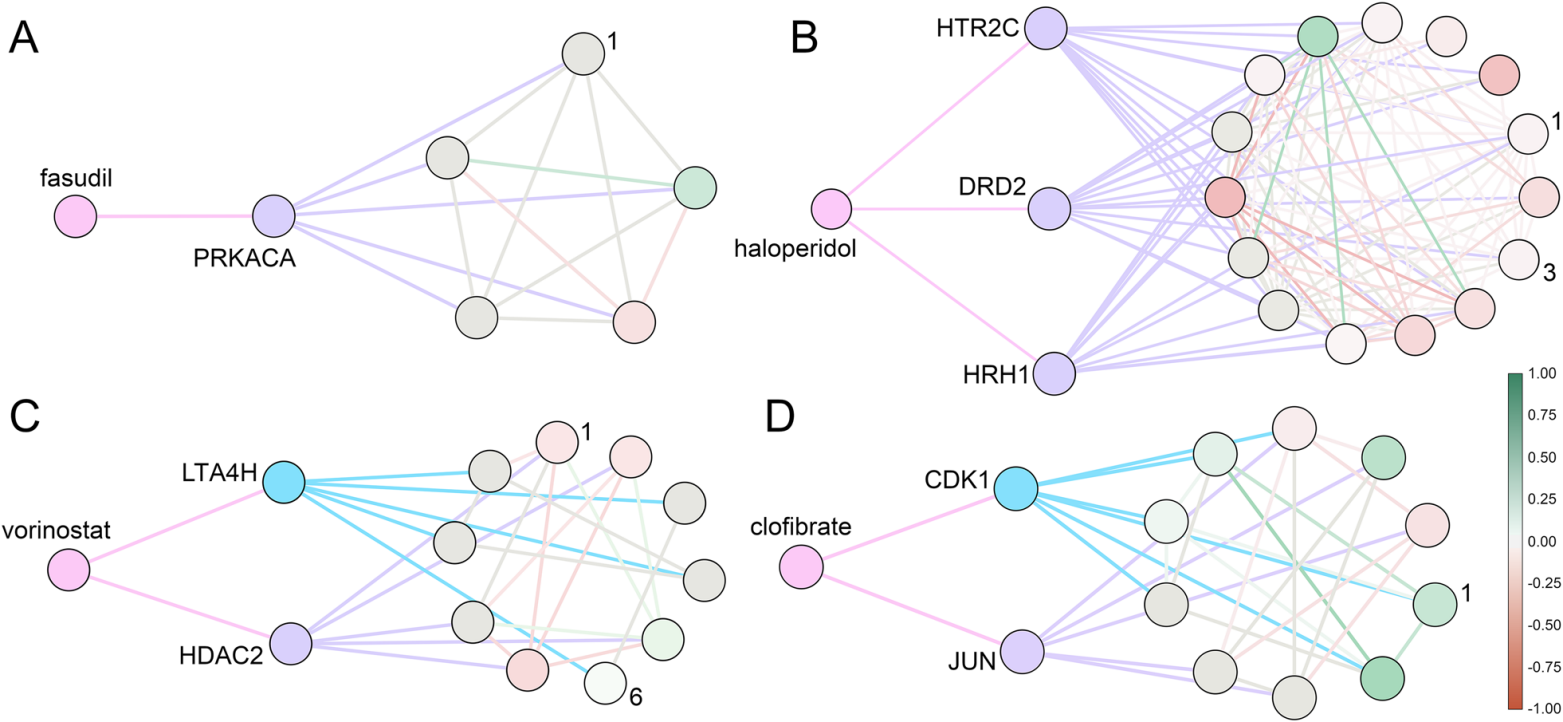
Examples of drug targets and their local network environment. **A** Fasudil interacting with cAMP-dependent protein kinase catalytic subunit α (PRKACA) further connected to AKAP1, PRKR2A, PRKR2B, PRKR1A, and PRKR1B. **B** Haloperidol interacting with 5-hydroxytryptamine receptor 2 C (HTR2C), dopamine D_2_ receptor (DRD2), and histamine H_1_ receptor (HRH1) along with a local network of connected proteins (NPSR1, PENK, ADRA2A, GNAQ, GRPR, TAC1, BDKRB2, KNG1, GRM5, HCRT, ADRB2, NTS, CCK, NCS1, and CDH1). **C** Vorinostat interacting with histone deacetylase 2 (HDAC2) and leukotriene A4 hydrolase (LTA4H) along with a local network of connected proteins (SIN3A, EZH2, LTC4S, PGM1, MBD2, ALOX5, RBBP7, RBBP4, ALDOA, and 1DH1). **D** Clofibrate interacting with transcription factor AP-1 (JUN) and cyclin-dependent kinase 1 (CDK1) along with a local network of connected proteins (CKS2, UBE2C, MAPK8, MAPK9, ANAPC4, CCNB2, AURKA, FOS, MAPK10, and ATF3). Drugs are colored in salmon, whereas targets are purple and off-targets are cyan. Nodes in local networks are colored according to their differential gene expression between drug-treated and untreated cells (green—upregulated, red—downregulated) and ordered clockwise starting with the most upregulated protein, labeled 1

Classified as an investigational small molecule according to DrugBank, fasudil is a potent RhoA/Rho kinase inhibitor used to treat carotid stenosis [52] and cerebral vasospasm [53]. cAMP-dependent protein kinase catalytic subunit α (PRKACA) is another important target of fasudil. Figure 9 A depicts the interaction of fasudil with PRKACA and a sub-network of PRKACA containing five other proteins, AKAP1 (labeled 1 in Fig. 9 A), PRKR2A, PRKR2B, PRKR1A, PRKR1B. Among these neighbor proteins, A-kinase anchor protein 1 (AKAP1) is a cardioprotective protein acting as a scaffold to recruit protein kinase A to the outer membrane of mitochondria [54]. It is important to note that fasudil has a protective effect on cardiac mitochondrial function and structure in rats with induced type 2 diabetes [55]. GraphDTI predicted an interaction between fasudil and PRKACA with a high score of > 0.99 across multiple cell lines, including a kidney cell line HA1E with a confidence of 0.9997. Indeed, the catalytic subunit α of bovine cAMP-dependent protein kinase has been co-crystallized with fasudil with a *K*_d_ of 5.7 µM [56]. Further, dimethylfasudil, an analog of fasudil, exhibits an inhibitory effect on HA1E cells overexpressing Myc proto-oncogene protein [57], which was shown to directly regulate the transcription of cAMP-dependent protein kinase catalytic subunit β [58].

### Polypharmacology of haloperidol

Haloperidol, a potent antagonist of dopamine receptors and the first-generation antipsychotic drug [59], is used to treat schizophrenia, Tourette syndrome, acute psychosis, and other behavioral problems [60]. According to DrugBank, molecular targets of haloperidol other than dopamine receptors, are histamine, serotonin, adrenergic, and sigma non-opioid intracellular receptors [16]. Figure 9B shows three targets of haloperidol, histamine H_1_ receptor (HRH1), 5-hydroxytryptamine receptor 2 C (HTR2C), and dopamine D_2_ receptor (DRD2) along with a local network of interacting proteins. Histamine H_1_ receptor interacts with TAC1, BDKRB2, KNG1, HCRT, and GRPR, whereas 5-hydroxytryptamine receptor 2 C interacts with NPSR1 (labeled 1 in Fig. 9B), GNAQ, GRM5, CCK, and NTS. A long-term treatment with haloperidol was found to upregulate the mRNA expression of neuropeptide S receptor (NPSR) in rat brain supporting the involvement of neuropeptide S in the pathophysiology of psychiatric disorders [61]. Among the network neighbors of dopamine D_2_ receptor, ADRA2A (labeled 3 in Fig. 9B), CDH1, NCS1, ADRB2, and PENK, ADRA2A was shown to weakly associate with haloperidol [62]. Based on the chemical structure of haloperidol and the sequence, structural, and network information for dopamine D_2_ receptor, histamine H_1_ receptor, and 5-hydroxytryptamine receptor 2 C, GraphDTI predicted their interactions with haloperidol with a high confidence of > 0.99 in multiple cell lines. Indeed, the binding affinities of haloperidol to these G-protein coupled receptors in terms of the inhibitory constant *K*_i_ are 2, 3000, and 5000 nM, respectively [63].

### Repositioning of vorinostat through off-target binding

Vorinostat is a hydroxamic acid-based inhibitor of histone deacetylases (HDAC) class I, II, and IV having antiproliferative effects against solid and hematologic cancers [64]. Figure 9C shows an interaction between vorinostat and histone deacetylase 2 (HDAC2) along with its sub-network comprising several proteins, SIN3A (labeled 1 in Fig. 9C), RBBP7, RBBP4, MBD2, and EZH2. Many of these proteins and the downstream signaling are affected by vorinostat binding to HDAC2. For instance, HDAC2 forms a complex with paired amphipathic helix protein Sin3a (SIN3A) acting as a corepressor for the p21 gene promoter, a negative regulator of the cell cycle progression [65]. Vorinostat disrupts this complex from binding to the p21 promoter by inhibiting the ING2 subunit binding to SIN3A, leading to the upregulation of the p21 gene [66]. GraphDTI predicted an interaction between vorinostat and HDAC2 with a high confidence of > 0.98 in pancreatic carcinoma cell lines, e.g., 0.9871 confidence for YAPC. Indeed, not only the inhibitory constant *K*_i_ of vorinostat measured against HDAC2 is 1 nM, but also the p21 gene blocking the G2/M-phase transition was found to be upregulated in pancreatic ductal adenocarcinoma cells [67].

Selected HDAC inhibitors were also found to inhibit leukotriene A4 hydrolase (LTA4H), a key enzyme in the biosynthesis of leukotriene B4 (LTB4), suggesting a possibility of their repositioning as anti-inflammatory agents in the treatment of idiopathic pulmonary fibrosis and acute lung injury [68]. Interactions between LTA4H and several other proteins, LTC4S, ALOX5 (labeled 6 in Fig. 9C), ALDOA, 1DH1, and PGM1, are also shown in Fig. 9 C. Among these neighbor proteins, polyunsaturated fatty acid 5-lipoxygenase (ALOX5) initiates the leukotriene synthesis from arachidonic acid in the LTB4 biosynthesis pathway [69]. GraphDTI predicted an interaction between vorinostat and LTA4H with a high confidence of > 0.99 across multiple cell lines. Experimentally determined half maximal inhibitory concentration (IC_50_) values for vorinostat and its analog M344 against LTA4H are 7.6 µM and 0.68 µM, respectively [68]. It is noteworthy that GraphDTI predicted no direct interaction between vorinostat and 5-LOX with low scores across multiple cell line ranging from 0.50 to 0.59. Indeed, experiments showed that vorinostat and its analog M344 are inactive against 5-LOX with a high IC_50_ of > 50 µM [68]. This case study demonstrates that DTIs predicted by GraphDTI can potentially suggest novel opportunities for drug repositioning.

### Off-target side effects of clofibrate

Clofibrate belongs to the hypolipidemic fibrate group of agents whose primary function is to increase the level of high-density lipoprotein and decrease the levels of low-density lipoprotein and triglycerides in plasma through the activation of peroxisome proliferator-activated receptor α (PPARA) [70]. The elevated expression of PPARA in the presence of clofibrate regulates mitochondrial and peroxisomal gene expression, which are involved in fatty acid metabolism in different tissues such as liver, brain, heart, kidney, adipose tissues, and intestine [70]. Fibrate-induced PPARA antagonizes various transcription factors, AP-1, STAT, and NF-κB, regulating inflammatory genes [71]. Through this repression, fibrate drugs modulate the anti-inflammatory response in the progression of atherosclerosis, a vascular inflammatory disease [71, 72]. Figure 9D shows the interaction between clofibrate and transcription factor AP-1 (JUN), predicted by GraphDTI with a high confidence of > 0.95 across various types of cell lines, and the corresponding sub-network of proteins interacting with JUN, including MAPK9, FOS, ATF3, MAPK8, and MAPK10. One drawback of fibrate drugs is that induced PPARα triggers the immediate early expression of growth regulatory genes, c-Jun, c-Fos, JunB, and NUP475 in liver, promoting tumor progression [73]. In addition, treatment with clofibrate increases β-oxidation of long-chain fatty acids and oxidative stress in rodent liver by producing hydroxyl radicals leading to hepatocellular toxicity [74].

GraphDTI also predicted an interaction between clofibrate and cyclin-dependent kinase 1 (CDK1) with a confidence of > 0.99 across multiple cell types. Interestingly, not only CDK1 is one of the cell proliferation markers, but also experiments conducted on homogenized liver from male rodents treated with clofibrate showed that the amount of CDK is significantly higher compared to untreated cells [74]. Figure 9D also depicts proteins interacting with CDK1, including CKS2 (labeled 1 in Fig. 9D), AURKA, CCNB2, UBE2C, and ANAPC4. Among these neighbors, cyclin-dependent kinase regulatory subunit-2 (CKS2) shows a higher expression in various hepatocellular carcinoma tissues [75]. According to these findings, the mechanism of hepatotoxicity of clofibrate may involve a putative interaction with CDK1, showing that interactions detected by GraphDTI can potentially reveal novel mechanisms of drug side effects.

## Conclusions

In this study, we developed a graph-based deep learning method, GraphDTI, to accurately predict DTIs from multiple heterogeneous data. In contrast to conventional feature-based DTI prediction algorithms usually employing features derived only from drug chemical structures and target protein sequences, GraphDTI utilizes other types of biological data. In addition to sequence embeddings, feature vectors also include structural, evolutionary, and physicochemical characteristics of ligand binding sites in the target proteins. Moreover, rather than focusing on a single interaction between a drug and a target, the information extracted from the human PPI network integrating drug-perturbed gene expression profiles of multiple proteins captures the system-level effects of a drug treatment. In order to avoid the curse of dimensionality, GraphDTI employs a state-of-the-art feature selection procedure. Interestingly, the optimized feature vectors not only yield a more robust performance, but also the analysis of the input vector composition demonstrates that the additional information on binding sites and the local network environment is vitally important to accurately predict DTIs.

Most studies focused on benchmarking algorithms to detect DTIs utilize random-split protocols, in which individual instances are randomly assigned to cross-validation folds. In this study, we devised a cluster-based protocol to assign instances into folds minimizing similarities between training and validation subsets. Comparative benchmarks utilizing random-split and cluster-based cross-validation demonstrate that the performance of many DTI predictors is overestimated when the former protocol is used. This is further confirmed in testing calculations against an independent dataset, in which only GraphDTI generalizes well to unseen data, while the performance of other methods is notably less satisfactory. It is also important to note that methods based on machine learning generally outperform traditional DTI prediction techniques utilizing inverse virtual screening with molecular docking.

Overall, GraphDTI offers a robust DTI prediction from multiple biological data for numerous applications in biomedicine, including the study of polypharmacological effects of drugs, the exploration of new opportunities for the repositioning of existing drugs to treat different conditions, and the investigation of drug side effects through off-target binding. GraphDTI is available as an open-source program from GitHub at https://github.com/Guannan1900/GraphDTI with the accompanying GraphDTI and PubChem BioAssay datasets accessible from the Open Science Framework at https://osf.io/ugvd9/.

## Materials and methods

### Drug-target interaction data

Experimentally determined data on DTIs were acquired from BindingDB, a web-accessible resource containing 1,881,721 interactions formed by 833,792 small molecules and 7548 target proteins [76]. As a positive DTI set, we selected 204,542 BindingDB interactions between 738 human proteins and 155,986 small molecules having identifiers in ChEMBL, a manually curated database of bioactive compounds with drug-like properties [77]. A negative DTI set comprises those combinations of drug-protein pairs, for which no similar pairs are present in the positive set. A similar pair is defined as the combination of a drug, whose chemical similarity measured by a Tanimoto coefficient (TC) [78] is ≥ 0.4, and a protein with a global sequence identity of ≥ 40 %. TC values for drug molecules were calculated with the kcombu program [79], whereas protein sequence identities were computed with the Needleman-Wunsch algorithm [80]. Because of a prohibitively large number of pairwise similarity calculations for the entire collection of 155,986 small molecules having ChEMBL identifiers, only a random subset of 10,000 compounds uniformly covering the chemical space were used to construct the negative DTI set. This set contains 3,745,178 negative interactions formed by 10,000 small molecules and 375 target proteins.

### Protein-protein interaction network

The STRING database comprises the protein-protein interaction data for 5090 organisms, including 11,355,804 interactions in the human proteome formed by 19,354 proteins [81]. Experimentally discovered and/or computationally inferred PPIs in STRING are annotated with confidence scores ranging from 150 to 999 with higher scores corresponding to more confident interactions between two proteins. From the initial set of DTIs, we selected only those interactions involving human proteins present in the STRING database (NCBI Taxonomy ID: 9606).

### Differential gene expression

Drug-perturbed gene expression profiles were obtained from the next-generation Connectivity Map (CMap) [18]. This resource comprises data collected for 107,404 combinations of 41 cell lines and 1797 small molecules, most of which were tested at six different concentrations, 0.04, 0.12, 0.37, 1.11, 3.33 and 10 µM. Each measurement is assigned a unique signature identifier containing the expression levels of 12,329 genes in terms of level 5 moderated *Z*-scores (MODZ). From the CMap database, we selected 30,461 combinations of 30 cell lines and 462 small molecules present in the initial set of DTIs compiled with BindingDB and mapped to STRING. Based on our experience, this data size may be too small for supervised machine learning, which could potentially lead to overfitting problems. On that account, we augmented the data to increase the number of DTI instances according to the biological knowledge.

### Knowledge-based data augmentation

Chemically related drugs typically share common binding profiles and can have similar clinical effects. For instance, several drugs having a TC of ≥0.8 with antihypertensive drug enalapril were shown to reduce high blood pressure and prevent heart failure [82]. At high concentrations, the transcriptomic profiles of chemically similar drugs with a TC of ≥ 0.85 tend to be similar as well [83]. Capitalizing on these observations, we developed a data augmentation protocol to significantly increase the size of the GraphDTI dataset. Specifically, for those BindingDB compounds having no data in CMap, we assigned gene expression profiles from the most similar molecules with a TC of ≥ 0.85 and at the highest tested concentration. Drug similarity searches for data augmentation were conducted using molecular fingerprints generated with Open Babel [84]. The final GraphDTI dataset comprises 326,139 positive instances involving 3618 drugs, 421 proteins, and 7590 signature identifiers, and 326,188 negative instances involving 236 drugs, 358 proteins, and 1541 signature identifiers. In terms of the number of unique drug-target pairs, the positive subset contains 10,977 pairs and the negative subset contains 79,376 pairs, totaling 90,353 drug-target pairs in the GraphDTI dataset.

### Unseen data for independent testing

In order to properly evaluate the generalizability of DTI predictors employing machine learning, an independent test dataset was compiled from the PubChem BioAssay database [51]. First, we selected those drugs from CMap that are not present in the BindingDB database, thus not included in the GraphDTI dataset. Mapping these compounds to the PubChem BioAssay database identified 389,076 experimentally tested drug-target combinations involving 195 drugs and 2152 proteins. Positive and negative subsets were constructed based on the bioassay outcome, i.e., those pairs annotated as “active” were considered as positive interactions, whereas “inactive” pairs were taken as negative interactions. After mapping drug-target pairs to CMap, the positive subset comprises 14,588 instances involving 51 drugs, 151 proteins, and 3248 signature identifiers, and the negative subset contains 58,714 instances involving 82 drugs, 47 proteins, and 3291 signature identifiers. The negative subset was randomly down-sampled to 14,588 instances involving 82 drugs, 47 proteins, and 2988 signature identifiers. The final PubChem BioAssay dataset for independent testing comprises 29,176 balanced instances, which are considered unseen data, viz. not present in the GraphDTI dataset and prepared using a different data source.

### Graph-based features for machine learning

For each target protein, an undirected, weighted subgraph is constructed according to the human PPI network from the STRING database [81]. The weights of edges are calculated as the reciprocal value of the confidence score between two interacting proteins. The graph distance between the target node and other nodes in the network is defined as the sum of the weights along the shortest path between these two nodes computed with Dijkstra’s algorithm [85]. Next, nodes are ranked in an ascending order according to their graph distances to the target node and then a fixed number of top-ranked nodes are selected to create a sub-graph centered on the target. This procedure ensures that the local network environment for each target protein has exactly the same dimension and comprises only those proteins connected through a relatively few, highly confident biological interactions according to STRING. Node features include the differential gene expression and the distance to the target node. Finally, Graph2vec is employed to learn the distributed representation of each subgraph [86]. This neural framework considers the input subgraph as a document and utilizes the Doc2Vec mechanism [87] to compute a 300-dimensional feature vector for the target protein based on its biological network environment.

### Molecular features for machine learning

Graph-based features are combined with molecular features to learn the representations of drug chemical structures, target protein sequences, and the physicochemical properties of drug binding sites. Drug features are extracted with Mol2vec, a natural language processing (NLP) model utilizing the Doc2Vec mechanism [88]. This approach considers chemical substructures covering all available chemical matter as the corpus of words and chemical compounds as sentences. The vector representations of protein sequences are computed with another NLP-based model, ProtVec, employing a Skip-gram neural network [89]. Another valuable data to infer DTIs are the representations of drug binding sites in target proteins. This information is computed with the Bionoi-AE [90], which first converts binding pockets identified in target proteins with *e*FindSite [91, 92] into Voronoi diagrams, and then generates latent vectors encoding the structural, evolutionary, and physicochemical features of drug binding sites. The default lengths of feature vectors in GraphDTI are 300 for Mol2vec and ProtVec, and 512 for Bionoi-AE.

### Multilayer perceptron architecture

GraphDTI utilizes the MLP, a classical feedforward neural network consisting of an input layer, two hidden layers, and an output layer, as the DTI classifier. The output of the $n$-th layer, ${L}_{n}$, in the MLP model is expressed as [93]:

1 $${L}_{n}={}_{n}({W}_{n}{L}_{n-1}+{b}_{n})$$

where ${W}_{n}$ is a weight matrix for the connections from the $(n-1)$-th layer to the $n$-th layer, ${b}_{n}$ are biases for neurons in the $n$-th layer, and ${\sigma }_{n}$ is the activation function in the $n$-th layer. The input layer in GraphDTI contains 400 neurons, both hidden layers have 128 neurons, and the output layer is composed of 2 neurons returning classes probabilities. The rectified linear unit (ReLu) function [94] is used as the activation function in all layers except for the output layer utilizing the softmax activation function [95]. The stochastic gradient descent (SGD) optimizer [96] and the cross-entropy loss function [97] are included in order to help the model learn effectively. GraphDTI uses the batch size of 32, the learning rate for the SGD optimizer of 0.0001, and the L2 weight decay of 0.00001. We found empirically that 30 epochs are sufficient for the model training to converge.

### Feature selection

Permutation feature importance is a widely used method for feature selection to help avoid the curse of dimensionality in deep learning [98]. This technique is applied against an independent testing dataset since it is important to evaluate the generalizability of a machine learning model by measuring the performance on unseen data. In this study, we first assessed the accuracy score of the MLP model with original, 1412-dimensional feature vectors, denoted as ${S}^{ori}$. Next, we randomly shuffled a single feature $j$ across all instances, without changing any other features or labels, to calculate a permutated accuracy score, ${S}_{j}^{perm}$. The importance of feature $j$, ${I}_{j}$, is defined as [99]:

2 $${I}_{j}={S}^{ori}-{S}_{j}^{perm}$$

### Random-split cross-validation

*K*-fold cross-validation is often employed to evaluate the generalizability of machine learning models. During the cross-validation, the entire dataset is first divided into *K* subsets without repetitions and then *K*-1 subsets are used for training while the remaining subset is used to evaluate the model performance. This procedure is performed iteratively until each subset has been used as the evaluation set. In this study, a 10-fold cross-validation is employed with two different protocols to divide the training data into folds. In a random-split protocol, cross-validation folds are created by randomly assigning drug-target pairs to *K* subsets. In order to make the results reproducible, a fixed seed is used to generate a random number series.

### Cluster-based cross-validation

The overlapping data problem can be mitigated by creating cross-validation folds from distinct groups of training instances obtained by the clustering of drug-target pairs. In this study, we employed the *k*-medoids algorithm [100], a clustering method similar to the *k*-means algorithm, to partition the GraphDTI dataset into clusters minimizing distances between instances in the same cluster and maximizing the distances between instances belonging to different clusters. Data clustering was conducted with three distance measures for drug-target pairs. The first distance is the FMD, defined as a Euclidian distance for the combined drug features calculated with Mol2vec [88] and protein features calculated with ProtVec [89]. The second is the PMD [50] based on the TC [78] between drugs and the Template Modeling score (TM-score) [101] between proteins, ranging from 0 to $\sqrt{2}$. Mapping all 90,353 drug-target pairs in the GraphDTI dataset to a coordinate system in the Euclidean space with the PMD puts them in a circle with a radius of $\sqrt{2}$. Since this representation makes it difficult for common clustering algorithms, such as *k*-medoids and *k*-means, to work satisfactorily, we developed the following scaled version of the PMD:

3 $$scaled \, PMD= \frac{PMD}{\sqrt{2}-PMD}$$

The scaled PMD is used as the third distance to cluster the GraphDTI dataset with the *k*-medoids algorithm. The quality of clustering with different distance measures and a varying number of clusters is evaluated with the SC [49]. After the best distance measure and the optimal number of clusters are determined, the resulting clusters are randomly merged into 10 folds, which are then employed in the cluster-based cross-validation against the GraphDTI dataset.

### Other approaches to DTI prediction

Machine learning-based DTI predictors, EnsemDT, EnsemKRR, and RLS-Kron [46], were selected for comparative benchmarks against GraphDTI. Similar to the original publication, these methods were deployed with drug features calculated with Mol2vec [88] and protein features calculated with ProtVec [89]. Inverse virtual screening was conducted with the docking program AutoDock Vina [5]. Drug molecules were docked to binding pockets identified in target proteins with *e*FindSite [91, 92] using optimized docking parameters [102]. For each drug molecule, all proteins were ranked based on the binding energies computed by Vina and the top-ranked molecules were predicted as the targets.

## Data Availability

GraphDTI is available at https://github.com/Guannan1900/GraphDTI. GraphDTI and PubChem BioAssay datasets are accessible at https://osf.io/ugvd9/.

## Abbreviations

AKAP1: A-kinase anchor protein 1
ALOX5: Polyunsaturated fatty acid 5-lipoxygenase
AUC: Area under the curve
CDK1: Cyclin-dependent kinase 1
CKS2: Cyclin-dependent kinase regulatory subunit-2
CMap: Connectivity Map
CNN: Convolutional neural network
DBN: Deep belief network
DNN: Deep neural network
DRD2: Dopamine D_2_ receptor
DTI: Drug-target interaction
FMD: Feature match distance
FPR: False positive rate
HDAC2: Histone deacetylase 2
HRH1: Histamine H_1_ receptor
HTR2C: 5-Hydroxytryptamine receptor 2C
IC_50_: Half maximal inhibitory concentration
IVS: Inverse virtual screening
JUN: Transcription factor AP-1
KRR: Kernel ridge regression
LINCS: Library of Integrated Network-based Cellular Signatures
LR: Local radiality
LSTM: Long short-term memory
LTA4H: Leukotriene A4 hydrolase
LTB4: Leukotriene B4
MODZ: Moderated *Z*-scores
MSE: Mean squared error
NLP: Natural language processing
NPSR: Neuropeptide S receptor
PMD: Perfect match distance
PPARA: Peroxisome proliferator-activated receptor alpha
PPI: Protein–protein interaction
PRKACA: CAMP-dependent protein kinase catalytic subunit α
ReLu: Rectified linear unit
ROC: Receiver operating characteristic
SC: Silhouette coefficient
SGD: Stochastic gradient descent
SIN3A: Paired amphipathic helix protein Sin3a
TC: Tanimoto coefficient
TM-score: Template Modeling score
TPR: True positive rate
t-SNE: T-distributed stochastic neighbor embedding
